# Are Markers of Systemic Inflammatory Response Useful in the Management of Patients With Neuroendocrine Neoplasms?

**DOI:** 10.3389/fendo.2021.672499

**Published:** 2021-07-22

**Authors:** Elisa Giannetta, Anna La Salvia, Laura Rizza, Giovanna Muscogiuri, Severo Campione, Carlotta Pozza, Annamaria Anita LIvia Colao, Antongiulio Faggiano

**Affiliations:** ^1^ Department of Experimental Medicine, “Sapienza” University of Rome, Rome, Italy; ^2^ Department of Oncology, University Hospital 12 de Octubre, Madrid, Spain; ^3^ Endocrinology Unit, Department of Oncology and Medical Specialities, AO San Camillo-Forlanini, Rome, Italy; ^4^ Endocrinology Unit Department of Clinical Medicine and Surgery, University Federico II School of Medicine, Naples, Italy; ^5^ A. Cardarelli Hospital, Naples Department of Advanced Diagnostic-Therapeutic Technologies and Health Services Section of Anatomic Pathology, Naples, Italy; ^6^ Department of Clinical Medicine and Surgery, University “Federico II”, Naples, Italy; ^7^ Department of Clinical and Molecular Medicine, Endocrine-Metabolic Unit, Sant’Andrea University Hospital “Sapienza” University of Rome, Rome, Italy

**Keywords:** neuroendocrine neoplasms, neutrophil-lymphocyte ratio, platelet-lymphocyte ratio, PD-L1, early response, cytokines, VEGF

## Abstract

Given the increasing incidence of neuroendocrine neoplasms (NENs) over the past few decades, a more comprehensive knowledge of their pathophysiological bases and the identification of innovative NEN biomarkers represents an urgent unmet need. There is still little advance in the early diagnosis and management of these tumors, due to the lack of sensible and specific markers with prognostic value and ability to early detect the response to treatment. Chronic systemic inflammation is a predisposing factor for multiple cancer hallmarks, as cancer proliferation, progression and immune-evading. Therefore, the relevance of inflammatory biomarkers has been identified as critical in several types of tumours, including NENs. A bidirectional relationship between chronic inflammation and development of NENs has been reported. Neuroendocrine cells can be over-stimulated by chronic inflammation, leading to hyperplasia and neoplastic transformation. As the modulation of inflammatory response represents a therapeutic target, inflammatory markers could represent a promising new key tool to be applied in the diagnosis, the prediction of response to treatment and also as prognostic biomarkers in NENs field. The present review provides an overview of the pre-clinical and clinical data relating the potentially usefulness of circulating inflammatory markers: neutrophil-lymphocyte ratio (NLR), platelet-lymphocyte ratio (PLR), cytokines and tissue inflammatory markers (PD-1/PD-L1), in the management of NENs. (1) NLR and PLR have both demonstrated to be promising and simple to acquire biomarkers in patients with advanced cancer, including NEN. To date, in the context of NENs, the prognostic role of NLR and PLR has been confirmed in 15 and 4 studies, respectively. However, the threshold value, both for NLR and PLR, still remains not defined. (2) Cytokines seem to play a central role in NENs tumorigenesis. In particular, IL-8 levels seems to be a good predictive marker of response to anti-angiogenic treatments. (3) PD-1 and PD-L1 expression on tumour cells and on TILs, have demonstrated to be promising predictive and prognostic biomarkers in NENs. Unfortunately, these two markers have not been validated so far and further studies are needed to establish their indications and utility.

## Introduction

The physiopathological association between chronic inflammation and cancer has been established for a long time (1–3). Although chronic inflammatory milieu could contribute to the development of cancer, several studies reported that tumor itself could begin and keep an inflammatory process up. A change in a set of cytokines and chemokines has been reported in studies regarding stomach (4), liver (5, 6), lung (7), esophagus (8), breast (9), and prostate cancer (10). These findings could be of interest to identify not only potential pathogenetic mechanisms but also novel diagnostic/prognostic markers (11). In this view, recent studies analyzed the immunophenotypes of cancer cells and cancer stromal cells in terms of usefulness as prognostic factors, showing the prognostic values of podoplanin-positive cancer-associated fibroblasts (CAFs) for patients with high-grade neuroendocrine carcinomas (HG-NEC) of the lung (12).

An important hallmark of cancer is that it can escape immune attack; therefore, chronic cancer-related inflammation could be considered as an attempt of immunosuppression mechanisms mediated primarily by immature myeloid-derived suppressor cells to block the development of cancer (13, 14). The fascinating link between inflammation and the field of neuroendocrinology has also been evaluated (15, 16). A bidirectional action between neuroendocrine stimuli and macrophage function in the development of innate and adaptive immune responses was described (17), suggesting a potential involvement of inflammation in the development of neuroendocrine neoplasms (NENs).

Neuroendocrine cells can be over-stimulated by chronic inflammation, which leads to hyperplasia and neoplastic transformation (18).

Research efforts have shown that NENs of gastroentero- pancreatic tract (GEP-NENs) occur more frequently in the settings of chronic inflammation. Indeed, it was shown that enteroendocrine cells can be hyperstimulated by chronic inflammation, which leads to their hyperplasia and neoplastic transformation (19–21).

Despite the progress in the understanding of NEN molecular biology, we are still far from the identification of markers able to detect the tumor at an early stage as well as to predict disease relapse after treatments.

As the modulation of inflammatory response represents a therapeutic target, changes of inflammatory markers may potentially represent in the future new biomarkers, which beyond the RECIST criteria, could eventually be helpful in the follow up of patient with NENs treated with targeted therapies.

This review investigated a panel of inflammatory response markers apparently heterogeneous but sharing the feature to be readily available and inexpensive diagnostic and prognostic factors in NENs.

## Prognostic Value of Neutrophil-to-Lymphocyte Ratio and Platelet-to-Lymphocyte Ratio for patients with NEN

The recent advent of detecting systemic inflammation levels through non-invasive blood tests, has opened the possibility of studying inflammatory processes at baseline and monitoring the course of cancer disease, in order to stratify patients according to their prognosis and to achieve a personalized approach (22). In this context, two *ratios*, neutrophil-lymphocyte ratio (NLR, calculated as the neutrophil count divided by the lymphocyte count) and platelet-lymphocyte ratio (PLR, obtained by dividing the platelet count by the absolute number of lymphocytes), have demonstrated to be powerful biomarkers for patients with cancer (23, 24). Notably, both NLR and PLR, are non-invasive, rapid, simple to acquire and inexpensive markers, thus, they could have a potential for widespread clinical use.

High NLR has been associated with poor clinical outcome in several tumor types (25). The underlying mechanism has not completely been elucidated, so far. Preclinical studies have shown that neutrophilia, which is a direct expression of systemic inflammation, represses the cytolytic activity of immune cells, such as lymphocytes, activated T cells, and natural killer cells (26). Additionally, tumor-associated neutrophils (TANs) have been demonstrated to promote tumor progression acting as pro-angiogenic agents (27), by a high expression of different pro-angiogenic factors as vascular endothelial growth factor (VEGF), Interleukin 1 beta (IL-1β) and Integrin Subunit Beta 1 (ITGB1) (28). Several studies have also reported that TANs are associated with an elevated expression of matrix metallopeptidase-9 (MMP-9), favoring angiogenesis through the MMP-9-VEGF axis (28).

PLR has arisen as a useful marker of systemic inflammation, metabolic syndrome and prothrombotic state and it is regarded as a promising biomarker in cancer patients (24, 29). Alterations in PLR have also been associated with other markers of systemic inflammation, particularly with NLR. Even in this case, as for NLR, the molecular mechanism has not been fully understood yet. Platelets represent an essential storage for secreted growth factors (as VEGF or platelet-derived growth factor, PDGF). In that way, platelets play a key role in regulating tumor angiogenesis, cell proliferation, migration, and metastasis (30, 31).

Therefore, despite the encouraging data about the clinical relevance and prognostic implication of NLR and PLR as biomarkers in cancer patients, some limitations still exist. For instance, a unique cut-off value of these two inflammatory *ratios* has not been established. Another open issue is to determine the best timing for dosing NLR and PLR, given the dynamic nature of this measures that change over times and that could be altered in relation to the administration of systemic treatments or because of other clinical conditions (as sepsis and septic shock) (32).

### Clinical Evidence in NENs

To date, several studies have been published about the role of the two ratios, NLR and PLR, in NENs. The available data are summarized in **Table 1**.

**Table 1:** Prognostic values of Neutrophil-Lymphocyte Ratio (NLR) and Platelet-to-Lymphocyte Ratio (PLR) in NENs patients.

Author, year	Mean age	Primary site	Grade	TNM stage	Metastasis	NLR cut-off	PLR
*Salman T. et al.* (13)	56.7	GEP-NENs	1-2-3	1-2-3-4	Metastatic & Non-metastatic	**2.17**	**181.5**
						Pretreatment	Pretreatment
						NLR >2.17->shorter	PLR >181.5
						median PFS	->shorter median
						(11.1 months)	PFS (11.2 months)
						NLR ≤ 2.17	PLR ≤ 181.5
						->longer median PFS (22.2 months)	-> longer median PFS (21.9 months)
						p = 0.001	p = 0.001
*Cao L-L. et al.* (16)	58	Gastric NENs	1-2-3	1-2-3-4	Metastatic & Non-metastatic	**2.20**	/
						Preoperative NLR>2.20 correlates with a shorter reccurence time, RFS and OS (p<0.01)	
						NLR >2.20	
						associated with both liver metastasis and peritoneal metastasis (P < 0.05)	
*Arima K.et al.* (15)	58	Pancreatic-NENs	1-2-3	/	Metastatic & Non-metastatic	**2.40**	/
						Preoperative NLR>2.40 ->	
						shorter RFS (<0.05) shorter OS (<0.0001) and postoperative liver metastasis (p<0.0001)	
*Tong Z. et al.* (33)	54.4	Pancreatic-NENs	1-2-3	1-2-3-4	Metastatic & Non-metastatic	**1.40**	/
						Preoperative	
						NLR > 1.4-> shorter RFS (p < 0.05).	
*Zhou B. et al.* (34)	52.9	Pancreatic-NENs	1-2-3	1-2-3-4	Metastatic & Non-metastatic	**2.31**	**151.4**
						NLR > 2.31-> shorter OS (HR=4.907, p<0.001)	PLR> 151.4 -> shorter OS (HR=3.307, p=0.003)
						NLR > 2.31-> shorter DFS (HR=4.143, p<0.001)	PLR > 151.4 -> shorter DFS (HR=2.617, p=0.001)
*Zhou B. et al.* (35)	53	Non- functioning Pancreatic-NENs	1-2-3	1-2-3-4	Metastatic & Non-metastatic	**1.80**	**168.25**
						NLR > 1.80->	PLR > 168.25->
						shorter DFS (p=0.007)	shorter DFS (p<0.001)
*Okui M. et al.* (36)	68.8	LCNEC	3	1-2-3	Non metastatic	**1.7**	/
						NLR value > 1.7 -> shorter OS (HR 8.559, p = 0.011).	
*Gaitanidis A. et al.* (37)	52	Pancreatic-NENs	1-2-3	/	Metastatic & Non-metastati	**2.3**	**160.9**
						NLR> 2.3 -> shorter PFS	PLR > 160.9 -> shorter PFS
						(HR 2.53, p = 0.038)	(HR 5.86, p=0.023).
*McDermott SM. et al.* (38)	57	GEP, colorectal and lung NENs	1-2-3	4	Metastatic	**4**	/
						NLR>4-> shorter OS (p=0.005).	
*Zou J. et al*. (39)	65	GEP, colorectal and other NENs	1-2-3	4	Metastatic & Non-metastatic	**2.8**	/
						NLR> 2.8 ->	
						shorter OS (p = 0.03)	
*Panni RZ. et al.* (40)	57.5	Pancreatic-NENs	1-2-3	1-2-3	Non-metastatic	**3.7**	/
						NLR> 3.7 ->	
						shorter RFS (HR 1.79, p=0.01) and shorter OS (HR 2.04, p=0.01)	
*Harimoto N. et al.* (41)	61	Pancreatic-NENs	1-2-3	1-2-3	Non-metastatic	**3.4**	/
						NLR> 3.4 ->	
						shorter RFS (HR 31.75, p=0.03)	
*Pozza A. et al.* (42)	70, 66, 63.5	Foregut, Midgut and hindgut NEN	1-2-3	1-2-3-4	Metastatic & Non-metastatic	**2.6**	Not significant association with OS
						NLR> 2,6 ->	
						shorter OS (HR 4.71, p=0.02)	
*Zhou B. et al.* (43)	60	Pancreatic-NENs	1-2-3	1-2-3-4	Metastatic & Non-metastatic	**3.1**	/
						NLR> 3.1 ->	
						shorter DFS (p<0.001) and OS (p=0.002)	
*Zhou W. et al.* (44)	53	Pancreatic-NENs	1-2-3	1-2-3-4	Metastatic & Non-metastatic	**1.9**	/
						NLR> 1.9 ->	
						shorter RFS (p=0.046) and in OS (p=0.032).	

GEP, gastro-entero-pancreatic; HR, hazard ratio; NEC, neuroendocrine carcinomas; NENs, neuroendocrine neoplasms; PFS, progression free survival; RFS, recurrence free survival; OS, overall survival.

In 2016 the Izmir Oncology Group Study retrospectively investigated the prognostic role of baseline NLR and PLR in 132 GEP-NENs patients. The included patients were equally distributed according to grading (31.1% G1, 33.3% G2, 35.6% G3). Embryonic origin was foregut in 87 cases, midgut in 20 cases and hindgut in 25. Primary site was pancreas in 50 cases and gastro-enteric tract in 82. 62 were metastatic patients. NLR and PLR were significantly higher in high grade NENs (0.0001), in metastatic patients (0.0001) and in those of foregut origin (0.0001). Patients with pancreatic NENs had higher NLR and PLR compared to those with gastrointestinal NENs (0.0001). Finally, higher NLR and PLR were negatively associated to progression-free survival (PFS) (0.0001), while no overall survival (OS) data were provided (13).

Another study, by Cao et al., evaluated the prognostic role of preoperative NLR in 147 gastric NENs (g-NENs) patients that underwent to radical surgery. Of them, 27 (18.4%) patients were gastric neuroendocrine tumors (g-NETs), 48 (32.7%) with gastric neuroendocrine carcinoma (g-NEC), and 72 (48.9%) with gastric mixed adenoneuroendocrine carcinoma (g-MANEC). Among these patients, 97 (66.0%) received adjuvant chemotherapy. Moreover, 147 healthy controls were enrolled. Significantly higher value of NLR was detected in patients with g-NENs compared to controls (P < 0.001). Furthermore, the NLR was an independent prognostic factor of relapse free survival (RFS) and OS (p<0.05 for both outcome measures), and, along with Ki67, positively correlated with liver metastases and negatively correlated with recurrence time (16).

One year later, a retrospective study aimed to evaluate the role of preoperative NLR as prognostic marker, was performed by Arima et al. (15). All the 58 pancreatic NENs patients included in the analysis, underwent curative pancreatic resection. Among these 58 patients, 46 were well differentiated G1 pancreatic neuroendocrine tumors (pNETs) and 31 were no-functioning tumors. The median NLR of all pNENs 58 patients was 2.18. A high preoperative NLR was significantly associated with higher tumor size (p= 0.0015) and grade 3 (p< 0.0001). In this analysis, the authors were able to identify a cut off value of NLR ≥2.4, that resulted associated to a worst OS (P = 0.0481) and RFS (P < 0.0001) and to an increased risk of postoperative recurrence (p= 0.0035).

In the same year, other three similar retrospective analysis were performed. All these three studies included G1, G2 and G3 pNENs. The first, included a population of 95 operated pancreatic NENs (33). Among these patients, 52 (54.7%) were G1 NET, 32 (33.7%) G2 NET, and 11 (11.6%) G3 NEC. A significant association was found between high NLR and advanced T stage, nodal metastasis, and advanced grade (p< 0.05 for all variables). High NLR value was confirmed as an independent prognostic factor for lymph-node metastasis by multivariate logistic regression (Hazard ratio (HR) 6.74; p=0.02)). NLR higher than 1.4 correlated with decreased RFS (p < 0.05). A second study, by Zhou B et al., evaluated both NLR and PLR in 172 patients with pNENs (34). 73 (42.4%) were G1 pNETs, 76 (44.2%) G2, and 23 (13.4%) G3 pancreatic NEC. 150 cases (87.2%) had stage I-II disease. 166 patients (96.5%), underwent R0 resection and 6 cases received palliative surgery (3.5%). In the study were enrolled also 172 healthy volunteers. A cut-off for NLR was identified as 2.31, for PLR was 151.4. NLR and PLR were significantly higher in the patients than in controls (all p<0.001). At univariate analysis an increased NLR and PLR correlated with advanced stage, high grade, and R1 resection (all p<0.05). High NLR or PLR had shorter OS (HR=4.907, p<0.001 and HR=3.307, p=0.003, respectively) and disease-free survival, DFS (HR=4.143, p<0.001 and HR=2.617, p=0.001, respectively) than patients with a low NLR or PLR). However, at multivariant analysis, only NRL remained significant as independent prognostic factor in terms of OS (HR=4.47, p=0.006) and DFS (HR=2.531, p=0.015). The third study, by Zhou et al., analyzed preoperative NLR and PLR in a population of 101 surgically removed pNENs (35). In this study, cutoff values were 1.80 for NLR and 168.25 for PLR. PLR and NLR were significantly higher in those patients with lymph-nodes metastases (p<0.05). At multivariable analysis, NLR (p= 0.017) correlated with lymph-nodes metastases. High NLR or PLR had shorter DFS (p=0.007 and p<0.001, respectively).

One year later, in 2018, a prospective study evaluating the role of NLR (calculated at baseline and preoperatively) and PLR (calculated at the time of enrollment for all patients, as well as preoperatively for patients who underwent resection with curative intent) in 97 pNENs, was published (37). The authors found that NLR higher than a cut-off values of 2.3 was a negative prognostic factor in terms PFS (HR 2.53, p = 0.038) and at multivariant analysis PLR > 160.9 resulted independently associated with reduced PFS (HR 5.86, p=0.023).

Another interesting retrospective study evaluated the role of NLR in a population of 26 completely resected large cells neuroendocrine carcinomas (LCNEC) (36). Notably, at multivariate analysis, a preoperative NLR value > 1.7 was confirmed as an independent prognostic factor for OS (HR 8.559, p = 0.011).

McDermott and colleagues, instead, investigated the prognostic value of NLR in 262 stage IV patients with liver metastases from different primary origins (GEP and pulmonary primary tumor), who were treated with transarterial chemoembolization (TACE) (38). As a result, pre-TACE NLR > 4 was associated with shorter OS (p=0.005). Additionally, pre-TACE NLR and 6-months post-TACE NLR resulted independently associated with OS on multivariant analysis (HR 1.4 p=0.030 and HR 1.7 0.007, respectively).

Another study focused on locally advanced and metastatic patients was performed in 2019 by the group of Zou and colleagues (39). In this case, were included 135 G1, G2 and G3 NENs of different primary origin. At univariate analysis, NLR > 2.8 correlated with OS (p=0.003), but the statistical significance was not confirmed at multivariant analysis.

Additionally, a year later in 2019, four retrospective analyses, which investigated the prognostic role of inflammatory markers in surgically removed pNENs patients were published. The first of them, included 620 non metastatic G1, 2 and 3 patients (40). With a cut-off of NLR of 3.7, the authors demonstrated a significative impact on RFS (HR 1.79, p=0.01) and OS (HR 2.04, p=0.01). The second study, by Harimoto N and colleagues, included 55 pNEN patients (41) and showed a negative prognostic role (in terms of RFS) for NLR>3.4, on univariate (HR 12.62, p<0.01) and multivariate analysis (HR 31.75, p=0.03). The third analysis was conducted on 64 operated pNENs (43). In this study, high NLR correlated with poor OS and DFS compared to patients with a low NLR score (p < 0.001). In the multivariate analysis, high NLR resulted an independent prognostic factor in terms of OS and DFS for pNENs of the head (p=0.002 and p<0.001, respectively). The fourth study, by Zhou W et al., included 174 pNENs (44). Even in this case, the prognostic role for NLR, with a cut-off of 1.9, was confirmed at univariant analysis, both in RFS (p=0.046) and in OS (p=0.032). However, multivariate analysis did not confirm that the NLR had an independent prognostic impact”.

Finally, a study on 48 G1, G2 and G3 NENs of different primary origins, but all surgically removed, was carried on by an Italian group (42). By a threshold value for NLR of 2.6, at the multivariable analysis high NLR was confirmed to have a significant impact on OS (HR 4.71, p = 0.02).

### Future Directions

Proinflammatory signals promote tumorigenesis and neoplastic progression, but their origins and downstream effects remain unclear. Given the pooled data of these studies about NLR and PLR, that confirm their role, these two inflammatory biomarkers could potentially represent innovative prognostic factors for NENs. In fact, both NLR and PLR are rapid, easy to measure, and cheap to obtain from routinary blood tests. In the analyzed studies both of them have been demonstrated to correlate with RFS and OS. Additionally, their combination with other markers such as proliferation index (ki67) and for example lymph node ratio, in order to obtain nomograms, has demonstrated to have a higher power to predict clinical outcomes of NEN patients (45). Polymorphonuclear neutrophils (PMN) also represent critical innate immune effector cells that either protect the host or exacerbate organ dysfunction by migrating to injured or inflamed tissues. Pathways including neuroendocrine and innate and acquired immune systems regulates PMN mobilization. In this view there is still no evidence of an accumulation of PMN in the NENs, but this aspect deserves to be examined (46).

However, there are many limitations of these data and some open questions. First of all, almost all the studies considered are retrospective and the sample size is quite often little. Furthermore, both the cut-off value used, and the population included is highly heterogeneous. Unfortunately, considering these issues a strong recommendation to the direct application in the clinical practice of NLR or PLR, couldn’t be given. However, the data presented are promising and should be confirmed in further prospective study, given the striking need to find new biomarkers in the field of NENs in order to better stratify patients by prognosis and to improve the personalization of therapeutic strategy.

## Circulating Cytokines as Possible Biomarkers of Therapeutic Response in Patients With NEN

The key molecular links between inflammation and cancer involve the canonical nuclear factor kappa-light-chain-enhancer of activated B cells (NF-kB) activation and Signal transducer and activator of transcription 3 (STAT3) pathways (47). NF-κB and STAT3 signaling pathways control genes necessary for angiogenesis (mainly VEGF) and influence the ability of tumor cells to invade and metastasize (48, 49). As a rule, most proinflammatory cytokines including tumor necrosis factor α (TNF-α), interleukin 6 (IL-6) and interleukin 17 (IL-17), produced by either the host immune system or the tumor cells themselves, promote tumor progression. In turn, pro-apoptotic TNF-related apoptosis-inducing ligand (TRAIL) and anti-inflammatory cytokines such as Interleukin 10 (IL-10) and transforming growth factor beta (TGF-β) usually lead to tumor suppression (50).

### Clinical Evidences in NEN

The role of cytokines (such as IL-1) in NENs differentiation has been demonstrated (51). Furthermore, Interleukin 2 (IL-2) has an established role in the regulation of the neuroendocrine system and in gastrointestinal hormone synthesis and secretion (52). Although normal pancreatic cells do not express Interleukin 8 (IL-8), pNENs show increased expression of IL-8 and its receptors, especially C-X-C Motif Chemokine Receptor 2 (CXCR2) (53, 54). In low-grade pNENs, normal circulating Placental Growth Factor (PlGF) values are associated with better survival, while in low-grade small intestinal NENs (SI-NENs) is an independent prognostic factor for shorter time-to-progression (55). The overexpression of VEGF promotes the growth of human NENs in part through up-regulation of angiogenesis (56). Low-grade NENs can synthesize, store and secrete VEGF, while, in HG-NENs this process is inconstant and heterogeneous. This feature is part of the so-called “neuroendocrine paradox”: in pNENs the density of the vascular network reflects the rate of differentiation rather than of aggressiveness: most is the vascularization, less the aggressiveness, and more differentiated pNEN are the less angiogenic. In this view, a recent study analyzing 60 resected HG-NEC of the lung (37 LCNECs and 23 Small Cell Lung Carcinomas -SCLCs), revealed the presence of stromal cells within vascular invasion was not significant predictor for recurrence. This suggests that the roles of intravascular stromal cells in HG-NEC metastasis are less, raise an “alarm” against overemphasis of stromal cell-targeting therapy (57).

Then, there is a strong rationale for supporting the use of angiogenesis inhibitor in well differentiated rather than poorly differentiated NENs. By contrast HG-pNENs are particularly active in terms of angiogenesis, meaning endothelial cell proliferation and abnormal vasculature (58).

In this view, cytokines panel represents an interesting tool in NENs, needing for framing. Cigrovski Berkovic et al. proposed a model of different cytokine genotypes and corresponding high serum values that regulate GEP-NEN etiopathogenesis (19).

Finally, Pavel et al. (59) showed that the circulating levels of VEGF and IL-8 are associated with tumor progression in patients with advanced NEC and might qualify as markers of prognosis and therapy control. Angiogenin and basic fibroblast growth factor (bFGF) levels do not correlate with tumor growth and with patient survival.

The prognostic utility of systemic inflammatory markers in NENs’ patients after therapy is still debated. The first-in-human trial of sunitinib (SUN) (60) included an analysis of plasma levels of VEGF and its soluble receptor, sVEGFR-2, of twenty-eight cancer patients (among them 4 patients were NENs), both pretreatment and after 28 days of treatment. VEGF concentrations increased slightly during the first month of SUN, while the plasma mean sVEGFR-2 decreased, demonstrating a targeted effect of the drug. Comparable findings were observed in another study on patients with metastatic NENs (61). After 28 days of SUN administration, VEGF levels increased more than 3-fold over baseline in about half of all patients, while sVEGFR-2 and sVEGFR-3 levels decreased by ≥ 30% in about 60% and 70% of all patients, respectively. Levels returned to baseline after two weeks of therapy interruption. Furthermore, IL8 values raised 2.2-fold average by the end of SUN cycle 1, and a larger increase was proportional to the tumor size reduction. This increase in IL-8 levels during SUN treatment can represent a mechanism of drug resistance, as also reported by Huang (62) in renal clear-cell carcinomas (RCC) cell lines. In addition, Zurita et al. (63) report that, at four weeks of the first cycle, SUN treatment is associated with significant increases from baseline in VEGF, IL-8, and stromal cell-derived factor-1 (SDF-1a) (also known as C-X-C motif chemokine 12, CXCL12), and with reduction in sVEGFR-2 and sVEGFR-3 with no difference between 66 pNENs and 39 carcinoid tumors. No significant associations have been found between soluble protein levels and clinical benefit response or PFS in pNENs, while high sVEGFR-3 and IL-8 levels correlated with shorter PFS and shorter OS in carcinoid tumors. Additionally, recent data come from the Spanish prospective SALSUN clinical trial enrolling well-differentiated pancreatic neuroendocrine tumors treated with sunitinib (PMID: 30651923). In this study, two SNPs in the VEGFR-3 gene, rs307826 and rs307821, predicted lower OS, with HR 3.67 and with HR 3.84, respectively. IL-6 was associated with increased mortality: HR 1.06, and osteopontin was associated with shorter PFS: HR 1.087, independently of Ki-67 value. Furthermore, levels of osteopontin remained higher at the end of the study in patients considered non-responders: 38.5 ng/mL *vs.* responders: 18.7 ng/mL, p-value=0.039. Dynamic upward variations were also observed with respect to IL-8 levels in sunitinib-refractory individuals: 28.5 pg/mL at baseline *vs.* 38.3 pg/mL at 3 months, p-value=0.024. In the RADIANT-3 phase III randomized clinical trial (64), baseline and post-treatment VEGF, PIGF, bFGF, sVEGFR-1, and sVEGFR-2 values were investigated in advanced pNENs’ patients treated with everolimus (EVE) 10 mg/die. In relation to the placebo, EVE treatment leads a significant and progressive reduction in sVEGFR-2 and an early but not significant decrease in PIGF. No significant differences in circulating concentrations of VEGF or sVEGFR-1 were observed. These data suggest a possible antiangiogenic effect of EVE as consequence of mTOR inhibition.

With regard to somatostatin analogs and interferon, in 36 patients with metastatic or unresectable carcinoid tumors (65), treatment with PEG interferon + depot Octreotide was associated with a significant increase in plasma Interleukin 18 (IL-18) and a significant reduction in plasma bFGF. No significant changes in the same plasma cytokines were associated with bevacizumab + depot octreotide therapy. Bevacizumab therapy resulted in objective responses, reduction of tumor blood flow, and longer PFS in patients with carcinoid than PEG interferon treatment. Finally, eight patients with NENs present lower VEGF plasma levels and reduced VEGF mRNA levels and microvessel density in liver metastasis biopsy material after IFN-α treatment (66). **Table 2** summarized circulating cytokine trend in response to different treatment approaches.

**Table 2:** Circulating cytokines trend according to different treatments.

		Treatment	N° pts	Tumor types	Tumor site	Tumor stage	Author, Year
VEGF	↑	SUN	4	NEN	digestive system	advanced	Faivre S. (60)
					(1/4 rectum)		
VEGFR-2	↓	SUN	4	NEN	digestive system		
					(1/4 rectum)		
VEGF	↑	SUN	109	NEN	pancreas	advanced	Bello CL. (61)
sVEGFR-2	↓	SUN	109	NEN	pancreas		
sVEGFR-3	↓	SUN	109	NEN	pancreas		
IL8	↑	SUN	109	NEN	pancreas		
VEGF	↑	SUN	65	NEN	pancreas	advanced	Zurita AJ. (63)
			35	carcinoid	foregut, midgut, hindgut		
IL-8	↑	SUN	66	NEN	pancreas		
			36	carcinoid	foregut, midgut, hindgut		
SDF-1a	↑	SUN	11	NEN	pancreas		
			10	carcinoid	foregut, midgut, hindgut		
sVEGFR-2	↓	SUN	65	NEN	pancreas		
			37	carcinoid	foregut, midgut, hindgut		
sVEGFR-3	↓	SUN	64	NEN	pancreas		
			34	carcinoid	foregut, midgut, hindgut		
PIGF	↓	EVE	393	NEN	pancreas	low – intermediate;	Yao JC. (64)
						advanced (unresectable or metastatic)	
sVEGFR1	=	EVE	393	NET	pancreas		
sVEGFR2	↓	EVE	390	NET	pancreas		
VEGF	=	EVE	393	NET	pancreas		
bFGF	=	EVE	393	NET	pancreas		
IL18	↑	PEG-IFN	22	carcinoid	foregut (3/22); midgut (11/22); hindgut (4/22); unknown (4/11).	low – intermediate;	Yao JC. (65)
		+				advanced (unresectable or metastatic)	
		OCT-LAR					
bFGF	↓	PEG-IFN	22	carcinoid	foregut (3/22); midgut (11/22); hindgut (4/22); unknown (4/11).		
		+					
		OCT-LAR					
IL18	=	BEV	22	carcinoid	foregut (3/22); midgut (13/22); unknown (6/11).	low – intermediate;	Yao JC. (65)
		+				advanced (unresectable or metastatic)	
		OCT-LAR					
bFGF	=	BEV	22	carcinoid	foregut (3/22); midgut (13/22); unknown (6/22).		
		+					
		OCT-LAR					
VEGF	↓	IFN-α	8	carcinoid	midgut	advanced (metastatic)	von Marschall Z. (66)

VEGF, vascular endothelial growth factor; sVEGFR, soluble vascular endothelial growth factor receptor; IL, interleukin; SDF-1a, stromal cell-derived factor 1; PIGF, placental growth factor; bFGF, basic fibroblast growth factor; SUN, sunitinib; EVE, everolimus; IFN-α, interferon α; PEG-IFN, peglyated interferon; OCT-LAR, depot long-acting octreotide; BEV, bevacizumab; NENs, neuroendocrine neoplasms; NET, neuroendocrine tumors; pts, patients.

### Future Perspectives

Cytokines seem to play a central role in NEN tumorigenesis. The observation that the modulation of IL-1 was positively related to a decrease in Chromogranin A (CgA) and a parallel increase in Carcinoembryonic antigen (CEA) secretion, suggest the key role of cytokines in NEN progression (51).

Together, the main results of the studies with large sample size suggest that the VEGF-pathway proteins and IL-8 are possible markers of prognosis and/or SUN treatment benefit in patients with GEP-NENs. Particularly, the IL-8 increase can represent a potential predictor of SUN response (61–63). VEGF, sVGEFR-2 and -3 changes can be new SUN’s biological activity biomarkers in NENs, confirming that SUN’s activity is mediated by the VEGF signaling pathway (60, 61, 63). Routine use of these circulating cytokines, in NEN patients’ clinical practice for SUN, is hopeful. Owing to the limited number of patients, further studies are needed to confirm the SDF-1α role in resistance to antiangiogenic SUN therapy.

A cross-talk between pro-inflammatory and angiogenic chemokines is described (67, 68). Interleukin-8 is an inflammatory cytokine upregulated in both cancer and chronic inflammatory diseases. Moreover, IL-8 is a chemokine that increases endothelial permeability during early stages of angiogenesis. IL-8 expression was inducible by hypoxia due to VEGF inhibition. In this view targeting both VEGF and IL8 it may be possible to achieve greater therapeutic efficacy.

As regard EVE treatment, except sVEGFR-2 and PIGF significant reduction, there are no significant differences in VEGF-pathway circulating proteins (64). These data suggest a possible antiangiogenic effect of EVE as a consequence of mTOR inhibition. Probably, other NENs biomarkers [such as Neuron-specific enolase (NSE) and CgA)] have a better prognostic value than the inflammatory cytokines in terms of survival and/or response to EVE treatment.

Given the strong rationale for using anti-angiogenic therapy for several tumors, basic and clinical research has shown a growing interest in investigating new related pathways (69). Tie2-expressing monocytes/macrophages (TEMs), Tie2 and VEGFR2 are highly expressed on stromal cells of the tumor microenvironment, especially on endothelial cells. Certain cancers, such as melanomas and gliomas, have been shown to lead to increased circulating Tie2+ monocytes and their recruitment to distal metastatic sites or anti-VEGF-treated gliomas (70). Recently an *in vitro* study proposed that modulation of Tie2+ proangiogenic macrophages through rebastinib, could possibly control tumor angiogenesis and lymphangiogenesis involved in cancer cell intravasation and metastasis in a model of pNENs (71). Our suggestion is that by identifying proinflammatory pathways in NENs we could extrapolate a set of prognostic markers useful in the management of NENs.

## Predictive and Prognostic Value of PD1 and PD-L1 for Patients With NEN

A key role in the immune-escape process is related to the interaction between programmed cell death protein 1 receptor (PD1) present on the surface of T lymphocytes and its ligand (PD-L1) on the surface of tumoral cells. PD-L1, by binding to PD-1, activate an inhibitory signal that avoids the destruction of cancer cells by host immune system.

In oncology, several compounds have been developed that act on this mechanism, and they are defined as Immune Checkpoint Inhibitors (ICIs). ICIs are monoclonal antibodies that bind to PD-1 (as Nivolumab and Pembrolizumab) or PD-L1 (as Atezolizumab, Avelumab and Durvalumab), respectively. The outcome of both bonds is to prevent the interaction between PD-1 and PD-L1 from blocking the T lymphocytes capable of attacking and eliminating tumour cells.

Immunotherapy acting through inhibition of PD1 and PD-L1 was firstly introduced with encouraging results in melanoma and non-small cell lung carcinomas (NSCLC), by using nivolumab and pembrolizumab, and now its use is widening in many other malignant tumors (72). To date, tissue expression of PD-L1 is tested by immunohistochemistry (IHC) and evaluated by microscopic assessment in all non-operable NSCLC, where the rate of expression in neoplastic cells can predict treatment response and its efficacy, indicating the place of pembrolizumab in the therapeutic algorithm (73, 74).

In the context of NENs, the potential efficacy of ICIs was investigated, at first, in HG, poorly differentiated NEC. HG-NEC are aggressive tumors, associated with a dismal prognosis (of approximately 10-12 months). The standard of care for this subgroup, still remains chemotherapy, which is associated with rapid but not long-lasting responses. Unfortunately, no targeted agents nor innovative approaches have been validated for NEC, so far. However, a rationale for the use of PD1 and PD-L1 inhibitors in this setting exists and it is represented by their high tumor mutational burden (TMB) (above all of SCLC), if compared to other type of cancer. Therefore, different ICIs have been tested in HG-NEC, confirming their activity. Some key examples are represented by skin Merkel cell carcinoma and SCLC, which almost always do not achieve durable remission with chemo- and radiotherapy, while the introduction of new therapies showed excellent and more durable responses (75, 76). In both cases, Merkel carcinoma and SCLC, ICIs have been approved and are currently used in daily clinical practice (77, 78).

However, the optimal selection of patients with HG-NEC as candidate for PD1 and PD-L1 inhibitors is still debated and immunohistochemical evaluation may not be alone satisfactory (76, 79–81).

On the other hand, for well-differentiated, low-grade NET, given their nature of more indolent tumors, with a very low TMB, and considering their relative favorable prognosis in the majority of cases, the potential activity of immune-checkpoint inhibition has not been established, so far. The current guidelines recommend surgery as the only curative treatment for early stages. For locally advanced inoperable or metastatic patients, depending on some essential clinic-pathologic features of each case (primary tumor localization, expression of somatostatin receptors on cell surface, ki67 value, presence of symptoms and tumor burden), the therapeutic armamentarium includes a great variety of active treatments as SSA (Octreotide or Lanreotide), targeted agents (i.e. the mTOR inhibitor EVE and the anti-angiogenetic drug SUN), peptide receptor radionuclide therapy (PRRT) or chemotherapy (82). All these treatments could be used individually or combined, and their sequence is decided case per case within the multidisciplinary-NEN dedicated tumor boards. However, the potential activity of ICIs in NET is still an open and challenging issue and hopefully the results of the studies currently ongoing in this field, could allow to define a role for this strategy.

### Clinical Evidence in NEN

As previously reported, a role for ICIs in HG-NEN is a promising therapeutic weapon. Unfortunately, no predictive biomarkers of response to anti-PD1/PDL1 therapy, have been established yet. It is well known that tissue expression and tissue localization (membrane of tumor cells or tumor-infiltrating immune cells, TILs) are both important for the access to the therapy (83). Therefore, the predictive value of PD-L1 expression in tumor cells and TILs by IHC has been investigated within several clinical trials for ICIs, for which different assays with specific IHC platforms were used. Of these, different PD-L1 IHC assays have been validated for the corresponding ICI. Not all laboratories, however, are equipped with dedicated platforms, and many laboratories are used to prepare house assays. Additionally, has been showed that the different available antibodies anti PD-L1 for IHC use are highly heterogeneous in their sensitivity to tumor cells expression or to TILs (83).

In this context, several authors have published results of PD-L1 tissue expression in lung NENs in the last years. The available data are summarized in **Table 3**. We will comment some of the most relevant papers, on lung NENs (84–90, 95, 96) as well as in Merkel cell carcinoma (92). In 2015, Schultheis and colleagues, were the first who investigated PD-1 and PD-L1 IHC expression in 61 SCLC. No expression in cancer cells was detected.

**Table 3:** Prognostic values of programmed cell death protein 1 receptor (PD1) and programmed death-ligand 1 (PD-L1) in NENs patients.

Author, year	Number of patients	Diagnosis	Tumor Grade	Metastasis	PD1/PDL1 and patients outcome
***Lung origin***					
*Fan et al.* (84)	80	22 NET,	1-2-3	Metastatic & Non-metastatic	The expression of PD1 in TILs was independently associated with OS (HR 0.367, p=0.001)
		48 SCLC,			
		10 LCNEC			
*Kim et al.* (85)	192	120 SCLC,	3	Metastatic & Non-metastatic	No relationship between PD-L1 expression on TCs and survival. Patients with PD-L1 expression on TILs had longer PFS than those without PD-L1 expression on TILs (11.3 *vs* 7.0 months, p=0.02)
		72 LCNEC			
*Kasajima et al.* (86)	242	57 NET, 127 SCLC,	1-2-3	Metastatic & Non-metastatic	For SCLC/LCNEC patients: PD-L1 positivity in TILs correlated with prolonged OS (p<0.01, HR 0.4)
		58 LCNEC			
*Inamura et al.* (87)	115	74 SCLC and 41 LCNEC	3	Metastatic & Non-metastatic	PD-L1 expression on TCs was an independent positive prognostic factor (p=0.0006, HR=0.29)
*Eichhorn et al.* (88)	76	LCNEC	3	Metastatic & Non-metastatic	PD-L1 expression on TCs and negative on TILs was associated with a worse prognosis (5-year TSS: 0% *vs* 60%; p<0.017)
*Xu Y. et al.* (89)	60	SCLC	3	Non-metastatic	PD-L1 expression on TCs was a negative independent prognostic factor for OS (HR=2.55, p =0.017)
*Sun C. et al.* (90)	102	SCLC	3	Non-metastatic	PD-L1 positive on TILs was associated with better RFS (p=0.004)
***Merkel cell carcinoma***					
*Wehkamp et al.* (91)	39	NEC	3	Metastatic & Non-metastatic	Shorter mOS for PD-1 positive patients (23.2 months *vs* 61.6 months, p=0.35); shorter mOS for PD-L1+ patients (PD-L1+ 24.7 *vs* PD-L1- 61.6 months, p = 0.86)
*Giraldo et al.* (92)	26	NEC	3	Metastatic	Higher density of expression on tumoral cells for PD-1 (median cells/mm2, 70.7 *vs* 6.7, p=0.03) and PD-L1 (855.4 *vs* 245.0, p=0.02) in responders *vs* not responders to pembrolizumab
**GEP origin**					
*Wang et al.* (93)	120	NENs	1-2-3	Metastatic & Non-metastatic	PD-L1 resulted an independent prognostic factor for OS
*Bösch et al.* (94)	244	NENs	1-2-3	Metastatic & Non-metastatic	PD-1 positive *vs* negative (44.5 months *vs* 53.8) and PD-L1 positive *vs* negative (46 months *vs* 51.9) had a negative impact on OS (p< 0.05, in both cases)

GEP, gastro-entero-pancreatic; HR, hazard ratio; LCNEC, large cell neuroendocrine lung carcinomas; NEC, neuroendocrine carcinomas; NENs, neuroendocrine neoplasms; NET, neuroendocrine tumors; NS, not specified; mOS, median overall survival; OS, overall survival; PFS, progression free survival; SCLC, small cell lung carcinomas; RFS, relapse free survival; TILs, tumor-infiltrating lymphocytes; TCs, tumor cells; TSS, tumor-specific survival; vs, versus.

In 2017, Inamura reported a PD-L1 positivity in 25 cases (21%) of a population of 74 SCLC and 41 LCNEC. The multivariant analysis confirmed PD-L1 expression on tumoral cells as an independent positive prognostic factor (HR=0.29; p=0.0006) in lung HG-NENs. In 2018, Eichhorn and colleagues performed a retrospective analysis of PD-L1 expression by IHC in tumoral cells and microenvironment, in a population of 76 LCNECs. The authors found positivity for PD-L1 (positivity was defined as the presence of PD-L1 in >1% of cells) only in tumor cells in 17 cases and only in the tumor microenvironment in 16 cases, while in 12 cases PD-L1 was positive in both cell types. A statistically significant difference in survival was observed comparing the cases with PD- L1 positive tumor/negative immune-cell infiltrate and PD- L1 negative tumor/positive immune-cell infiltrate, being the first associated with a worse prognosis (5-year Tumor-specific survival, TSS: 0% *vs.* 60%; p < 0.017). This observation was confirmed in 2019, by Xu Y, who reported that PD-L1 expression on tumoral cells was as an independent prognostic factor for OS (HR=2.55, p =0.017) in a population of 60 SCLC patients. The same conclusions came from a more recent study, published in 2020 by Sun C and his colleagues. This analysis included 102 surgically removed stage I, II and III SCLC.”. 40.2% and 37.3% of cases were detected to present a positivity on TILs for PD-1 and PD-L1, respectively. Only 3.9% of tumor cells resulted positive for PD-L1. TILs positive cases for PD-L1 presented better RFS (p=0.004). In the same direction, Fan Y. et al. demonstrated that the expression of PD1 in TILs remained independently associated with survival (HR, 0.367; p=0.001) in a population of 80 lung NENs (22 NET, 48 SCLC and 10 LCNEC).

**Figure 1:**
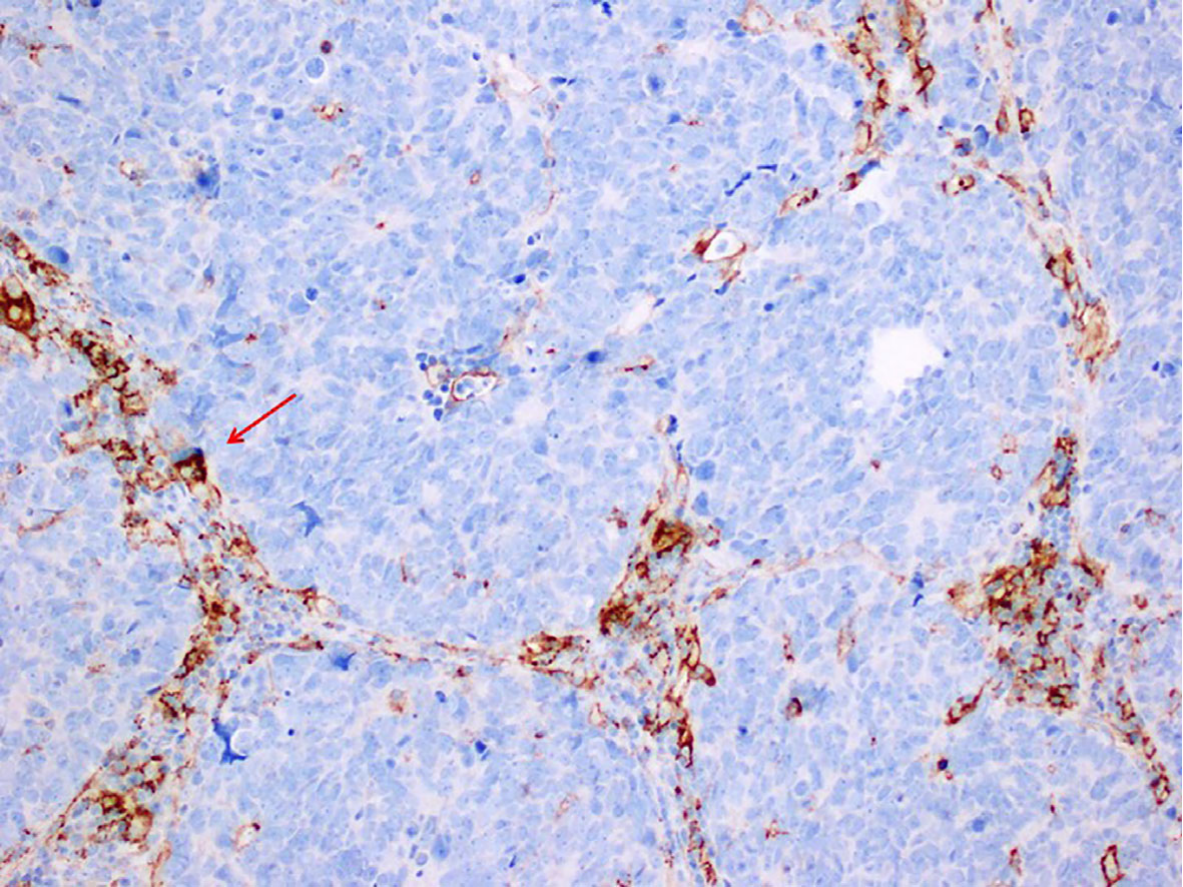
Immunohistochemistry stain, antibody anti PD-L1, SP263 VENTANA 200X MAGNIFICATION in a case of Small Cell Lung Carcinoma. PD-L1 positivity is limited to tumor infiltrating lymphocytes that lie between the nest of tumor cells, that are negative to PD-L1.

**Figure 1** shows a case of SCLC, followed at the Department of Advanced Diagnostic-therapeutic technologies and health services Section of Anatomic Pathology (A. Cardarelli Hospital, Naples, Italy*)*, where PD-L1 positivity was limited to TIL, while tumor cells were negative (**Figure 1** **)**.

Among Merkel cell carcinoma, in a very interesting study 39 patients were analyzed for immunohistochemical PD-1, PD-L1 and nerve growth factor (NGF) expression. These variables were correlated with clinic and pathological features, showing that PD-L1 and NGF are co-expressed on spindle cells in the microenvironment. Authors suggested that this co-expression might be a link of the microenvironment to the tropomyosin receptor kinase A (TrkA)-positive tumor cells, representing a critical mechanism for tumor growth and lack of response to anti-PD-1/L1 treatment, requiring to be investigated in further studies (91). Another study, by Giraldo et al, included 26 advanced Merkel cell carcinomas and investigated PD-1 and PD-L1 expression in order to determine their role as predictive biomarkers of response to ICIs. In this case, all the patients received treatment with Pembrolizumab. Higher density of expression on tumoral cells for PD-1 and PD-L1 were detected in responders versus not responders to the therapy (median cells/mm2, 70.7 *vs*. 6.7, p=0.03; and 855.4 *vs*. 245.0, p=0.02, respectively).

Additionally, PD-L1 expression has been also related to TMB (85) and tumor inflammation (86). Kim H.S. et al. found that the PD-1/PD-L1 pathway is activated in the microenvironment of pulmonary HG-NEN and correlated with a higher TMB, both in SCLC and LCNEC. Moreover, Kasajima et al. found an increased PD-L1 expression in TIL both in SCLC and LCNEC with a higher tumor associated inflammation and T cell CD8+.

Furthermore, in other HG NEN, beyond SCLC and Merkel cell carcinoma (that are the two fields in which ICIs have demonstrated their activity), PD-1 and PD-L1 are also under evaluation as potentially useful biomarkers.

Among GEP-NEN, only few studies about the evaluation of PD-L1 by IHC have been published so far. In 2017, Cavalcanti and colleagues, studied the expression of this tissue marker in 57 G1, G2 and G3 extrapulmonary-NENs (85, 97, 98). The authors found a significant correlation between PD-L1 expression by tumor cells and immune infiltrates and G3 of WHO classification (p=0.001), while it was not associated with gender, primary site, or number of metastatic sites. The next year, Lamarca et al., evaluated PD-L1 expression in 62 well-differentiated, G1 or G2 Si-NETs. PD-L1 was studied in tumoral cells as well as in TILs (85, 97, 98). PD-L1 expression was positive in 12.8% of cases and in 24.3% of TILs. PD-1 was expressed in 22.8% of TILs. Furthermore, the results obtained by IHC were confirmed with RT-qPCR. This technique detected higher expression levels of PD-L1 (p=0.007) and PD-1 (p=0.001) in samples positive by IHC compared to negative by IHC. In 2019, Wang and colleagues (93), investigated the positivity for PD-1/PD-L1 in 120 GEP-NENs. In this study, PD-L1 was expressed in 52.5% of the tumor cells, while PD-L1 was positive in 55.8% of TILs. At multivariate analysis, PD-L1 resulted an independent prognostic factor in this population. Additionally, Bösch and colleagues, included 244 pancreatic and G1, G2 and G3 SI-NEN patients (94). In this study, PD-1/PD-L1 were analysed on TILs, where a high PD-1 expression was demonstrated in 35 samples (16.1%), and a high PD-L1 expression was evidenced in 20 cases (8.7%). A significant negative impact on OS for PD-1 and PD-L1 positivity was demonstrated (p< 0.05, in both cases).

### Future Directions

The rationale of investigating PD-1 and PD-L1 expression in NENs is represented by the clinical need to find predictive biomarkers of response to ICIs. However, the role of PD-1 and PD-L1 tissue testing (in tumoral cells as well as in TILs) in defining the access to immunotherapy in NENs is still uncertain (87, 96).

The majority of the available supporting data are in the field of HG-NEN, as previously reported in detail. To date, skin Merkel cell carcinoma should be considered a paradigm for the efficacy of immunotherapy in NENs. However, PD-1 and PD-L1 tissue testing has not been validated as a fundamental predictive marker for patients selection (75). Also, in SCLC ICIs treatment has been approved, but even in this case the debate regarding predictive biomarkers of response is still an open issue (76). Among GEP-NEN, only a couple of studies have been carried on.Taking all the results together, PD-1 and PD-L1 expression appear to possibly have a role as negative prognostic biomarkers. However, further prospective studies, aimed to determine the epidemiology and the role as predictive or prognostic markers in NENs should be highly encouraged.

## Conclusions

NENs are a complex family of tumors, extremely heterogeneous in terms of primary origin of the tumor, tumor morphology (from well differentiated to poorly differentiated forms), proliferation index, clinical presentation and prognosis. To date, several treatments are available for NENs, including SSA, PRRT, targeted agents, chemotherapy, surgery and locoregional approaches. However, despite a clear role for inflammation in cancer and in NENs, only few immunotherapy agents have been approved (mainly in Merkel cell carcinoma and in SCLC) and above all no specific biomarkers capable of early predicting response to these agents, have been validated so far.

NLR and PLR and pro-inflammatory cytokines could represent a new tool for the early management of NENs. However, future studies adopting a prospective and matched study design need to confirm the role of inflammatory markers in NENs diagnosis, response evaluation, prognosis, and follow-up.

In conclusion, this panel of circulating inflammatory markers, correlated where possible with tissue markers, may be of utility if integrated in a cluster as biomarkers for targeted therapies response in clinical practice.

## Author Contributions

EG, AS, LR, GM, SC, and AF selected the issue, researched studies from databases, and independently analyzed published data. EG, AS, LR, GM, SC, CP, and AF contributed to the final version of the manuscript. EG, CP, and AF performed quality control checks on extracted data. AC verified the analytic method and supervised the planned systemic review of literature. SC conceived the figure extracting images from his analysis of a case. All authors contributed to the article and approved the submitted version.

## Funding

Ministerial research project PRIN2017Z3N3YC

## Conflict of Interest

The authors declare that the research was conducted in the absence of any commercial or financial relationships that could be construed as a potential conflict of interest.

## Glossary

**Table:**

bFGF	basic fibroblast growth factor
CAFs	cancer-associated fibroblasts
CEA	carcinoembryonic antigen
CgA	chromogranin A
DFS	disease-free survival
EVE	everolimus
g-NENs	gastric neuroendocrine neoplasms
g-NEC	gastric neuroendocrine carcinoma
g-MANEC	gastric mixed adenoneuroendocrine carcinoma
GEP	gastroenteropancreatic
HG-NEC	high-grade neuroendocrine carcinomas
HR	hazard ratio
ICIs	Immune Checkpoint Inhibitors
IL-1β	Interleukin 1 beta
IL-2	Interleukin 2
IL-6	Interleukin 6
IL-8	Interleukin 8
IL-10	Interleukin 10
IL-17	Interleukin 17
IL-18	Interleukin 18
IHC	immunohistochemistry
ITGB1	Integrin Subunit Beta 1
LCNEC	large cell neuroendocrine lung carcinomas
MMP-9	matrix metallopeptidase-9
NECs	neuroendocrine carcinomas
NENs	neuroendocrine neoplasms
NETs	neuroendocrine tumors
NF-κb	nuclear factor kappa-light-chain-enhancer of activated B cells
NLR	neutrophil-lymphocyte ratio
NSCLC	non-small cell lung carcinomas
NGF	nerve growth factor
NSE	Neuron-specific enolase
OS	overall survival
pNENs	pancreatic NENs
PNETs	pancreatic NETs
PlGF	Placental Growth Factor
PD-1	programmed cell death protein 1 receptor
PD-L1	programmed death-ligand 1
PDGF	platelet-derived growth factor
PFS	progression-free survival
PLR	platelet-lymphocyte ratio
PRRT	Peptide receptor radionuclide therapy
SCLC	small cell lung carcinomas
SSA	somatostatin analogues inhibitors
STAT3	signal transducer and activator of transcription 3
SDF-1a	stromal cell-derived factor-1
SUN	sunitinib
RCC	renal clear-cell carcinomas
RFS	relapse free survival
TANs	tumor-associated neutrophils
TACE	transarterial chemoembolization
TEMs	Tie2-expressing monocytes/macrophages
TGF-β	Transforming growth factor beta
TILs	tumor-infiltrating immune cells
TMB	tumor mutational burden
TNF-α	tumor necrosis factor
TRAIL	TNF-related apoptosis-inducing ligand
TRKA	tropomyosin receptor kinase A
VEGF	vascular endothelial growth factor
